# Shibasaburo Kitasato (1853-1931): Pioneer of Japanese Medicine and Global Immunology Innovator

**DOI:** 10.7759/cureus.68276

**Published:** 2024-08-31

**Authors:** Nobuo Okui

**Affiliations:** 1 Dentistry, Kanagawa Dental University, Yokosuka, JPN

**Keywords:** historical vignette, plague research, medical education, immunology, japanese medicine, bacteriology, serum therapy, shibasaburo kitasato

## Abstract

Shibasaburo Kitasato (1853-1931), a pivotal figure in modern Japanese medicine, made groundbreaking contributions to bacteriology and immunology. His achievements include pure cultivation of the tetanus bacillus and the development of serum therapy, which continue to influence modern immunology and vaccination strategies. Kitasato established the Institute for Infectious Diseases in Japan, the Kitasato Institute, played a crucial role in establishing the Keio University School of Medicine, and served as the first president of the Japan Medical Association. His international collaboration with Robert Koch and Emil von Behring elevated Japan's status in global medical research. Kitasato's research philosophy emphasizes clinical applicability and passionate pursuit of effective topics, inspiring the generation of prominent Japanese medical researchers. This study highlights Kitasato's life, work, and enduring impact on medical research, education, and healthcare administration. Kitasato's legacy, commemorated on the new 1000-yen banknote in 2024, continues to serve as an inspiration for contemporary medical professionals worldwide.

## Introduction and background

Shibasaburo Kitasato (1853-1931), widely recognized as the "father of modern Japanese medicine," made pioneering contributions to bacteriology and immunology that have profoundly shaped both Japanese and global medical history [1,2]. Born in Kumamoto prefecture, Kitasato's journey from a young martial arts enthusiast to a world-renowned bacteriologist exemplifies his dedication to science and medicine [1-3].

Kitasato's education began at the Kokuritsu Medical School in Kumamoto, where he was encouraged by Dr. Mansfeld to pursue a career in medicine [1-3]. His subsequent move to Tokyo Medical School in 1874 laid the foundation for his future research [1]. In 1885, Kitasato's career took a significant turn when he was sent to Germany to study under the renowned bacteriologist Robert Koch [1].

During his time in Germany, Kitasato achieved the following two major breakthroughs: the pure cultivation of the tetanus bacillus and the discovery of its antitoxin [4]. These achievements established his reputation as a brilliant researcher and laid the groundwork for the development of serum therapy [4]. His 1890 paper on anaerobic bacteria, "Zur Kenntniss der Anaëroben," further solidified his standing in the field of bacteriology [5].

Upon returning to Japan in 1892, Kitasato established the Infectious Disease Research Institute, which became a center for infectious disease research in Japan [1]. Later, in 1917, he played a key role in founding the Keio University School of Medicine, emphasizing the importance of practical education in medical training [1-3].

This paper aimed to explore Kitasato's life, his significant contributions to medicine, and their enduring impact on contemporary medical practice and research. As we approach the commemoration of Kitasato's legacy with his portrait on the new 1000-yen banknote in July 2024, it is an opportune moment to revisit the work of this remarkable scientist and its relevance to modern medicine.

## Review

Early life and education

During his early years, Kitasato was more interested in martial arts than academics. However, encouraged by his family, he entered the Kokuritsu Medical School in Kumamoto (later the Kumamoto University School of Medicine) [1]. It was here that he met the Dutch physician Dr. Mansfeld, who recognized Kitasato's potential and encouraged him to pursue a career in medicine [2].

In 1874, Kitasato moved to Tokyo to study at the Tokyo Medical School (later the Faculty of Medicine, University of Tokyo) [3]. This move laid the foundation for his future medical knowledge and research. His time at the Tokyo Medical School was crucial in shaping his understanding of modern medical practices and research methodologies [2,3].

German studies and breakthrough research

In 1885, after being employed by the Ministry of Home Affairs, Kitasato was sent to Germany on a government scholarship to study under the world-renowned bacteriologist, Robert Koch [1]. This period marked a significant turning point in Kitasato's career.

During his time in Germany, Kitasato made numerous contributions to bacteriology. He published a paper on the behavior of typhoid and cholera bacilli in acid and alkaline media, demonstrating his meticulous approach to research and his growing expertise in bacteriology [6].

Kitasato's most significant achievement came in 1889 when he succeeded in the first pure cultivation of the tetanus bacillus [7]. This breakthrough was followed by his discovery of the antitoxin for the tetanus toxin. These achievements not only established Kitasato's reputation as a brilliant researcher but also laid the groundwork for the development of serum therapy [8].

In collaboration with Emil von Behring, Kitasato published a groundbreaking paper on diphtheria and tetanus immunity in animals [9]. This work was fundamental to the development of serum therapy and marked a significant advance in immunology. The paper, titled "Ueber das Zustandekommen der Diphtherie-Immunität und der Tetanus-Immunität bei Tieren," is considered a cornerstone in the field of immunology [8,9].

**Figure 1:**
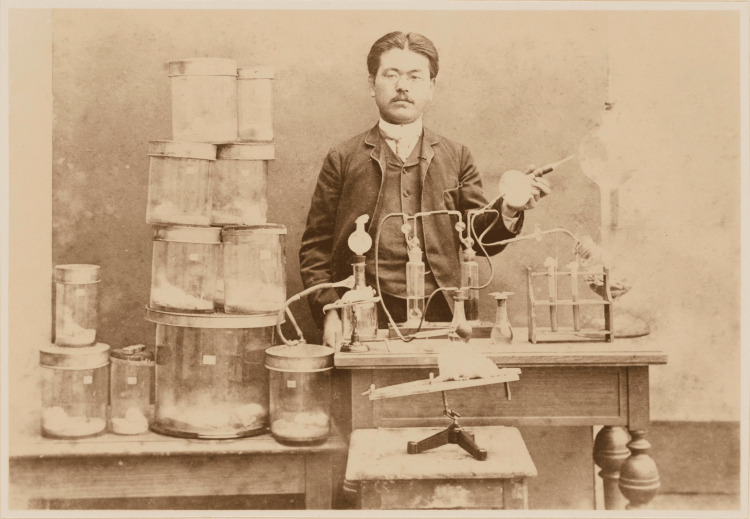
Dr. Shibasaburo Kitasato in his laboratory (Robert Koch's Institute for Infectious Diseases) during his studies in Berlin, Germany in 1889. Photo courtesy of the Kitasato Institute, Shibasaburo Memorial Museum. This image is in the public domain due to copyright expiration.

Figure 1 is a valuable portrait of Dr. Shibasaburo Kitasato. This photograph was taken during his study period in Germany. It captures Dr. Kitasato at the age of 37 years in 1889, immersed in his research. This year was pivotal for Dr. Kitasato, as he succeeded in the pure cultivation of the tetanus bacillus, paving the way for the establishment of serum therapy for tetanus.

The photograph depicts Dr. Kitasato amidst innovative experimental equipment he developed. At that time, it was believed impossible to cultivate pure tetanus bacteria cultures. However, Dr. Kitasato ingeniously created a special cultivation apparatus that used hydrogen to expel oxygen, exploiting the bacteria's anaerobic nature. He also developed the sealable "turtle-shell" Petri dish. These creative modifications to experimental tools demonstrate Dr. Kitasato's resourcefulness and dedication to advancing bacteriological research, challenging the scientific limitations of his era.

Establishment of the Infectious Disease Research Institute

Upon returning to Japan in 1892, Kitasato, with the support of Yukichi Fukuzawa, established a private Infectious Disease Research Institute [1]. This institute became the center of infectious disease research in Japan, with Kitasato serving as its first director. The following year, Kitasato founded Tsukushioka Sanatorium, a hospital specializing in tuberculosis treatment [1,9].

Kitasato's work in Japan continued to build on his research from Germany. He applied his knowledge of bacteriology and immunology to address pressing public health issues in Japan, including the study and treatment of infectious diseases such as plague and tuberculosis [9]. His institute played a crucial role in advancing medical research in Japan and establishing the country as a leader in the field of bacteriology [9].

Discovery of the plague bacillus and further contributions

The discovery of the plague bacillus during the 1894 Hong Kong plague outbreak is surrounded by controversy. Both Shibasaburo Kitasato and Alexandre Yersin reported the discovery of the bacillus almost simultaneously. Kitasato, a renowned Japanese bacteriologist, arrived in Hong Kong to conduct his research under challenging conditions. He successfully identified a bacterium in the blood of plague victims and initially claimed to have discovered the plague-causing organism, publishing his findings in the Lancet [10]. However, the bacterium Kitasato described differed in some aspects from the one Yersin reported, leading to significant debate within the scientific community [9]. Over time, it was Yersin's findings that gained wider acceptance, and the bacterium he identified was eventually recognized as the true causative agent of the plague, now known as Yersinia pestis [9]. Despite this, Kitasato's early work laid crucial groundwork and his efforts were instrumental in the broader understanding of the plague.

In addition to his work on the plague, Kitasato made significant contributions to the broader field of bacteriology. One of his most important achievements was his research on anaerobic bacteria. In 1890, Kitasato published a seminal paper titled "Zur Kenntniss der Anaëroben" ("Knowledge of Anaerobes") in Germany [5]. This study provided significant insights into the growth conditions and characteristics of anaerobic bacteria, which are microorganisms that thrive in environments devoid of oxygen. His research was pioneering at the time and further solidified Kitasato's reputation as a leading researcher in bacteriology [5,9]. The findings in this paper advanced scientific knowledge and provided practical implications for understanding various diseases and infections caused by anaerobic bacteria.

Kitasato's collaborative work with Emil von Behring on diphtheria and tetanus immunity in animals, published in 1890, was another landmark contribution [8]. This research laid the foundation for the development of serum therapy and marked a significant advance in immunology. Kitasato's work in these diverse areas of bacteriology and immunology continues to be referenced in scientific literature and remains a cornerstone in the study of infectious diseases, reflecting his lasting impact on the field.

Serum therapy: the pinnacle of Kitasato's scientific career

The development of serum therapy stands as the pinnacle of Kitasato's scientific career, representing his most significant and enduring contribution to medicine. This groundbreaking therapy, now considered one of the foundational treatments in modern medicine, emerged from the collaborative work between Kitasato and Emil von Behring in the early 1890s. Their seminal paper, "On the development of diphtheria immunity and tetanus immunity in animals," published in 1890, laid the foundation for serum therapy [8]. This work demonstrated that immunity against diphtheria and tetanus could be transferred from one animal to another, a revolutionary concept at the time.

Kitasato further expanded on this research with his comprehensive study on the tetanus toxin, "Experimentelle Untersuchungen über das Tetanusgift" (Experimental investigations on the tetanus toxin), published in 1891 [11]. This paper provided crucial insights into the nature of tetanus toxin and its neutralization, further advancing the field of serum therapy. The impact of their work was so profound that it led to the awarding of the first Nobel Prize in Physiology or Medicine to von Behring in 1901, with Kitasato's crucial role widely acknowledged in the scientific community [4,9].

**Figure 2:**
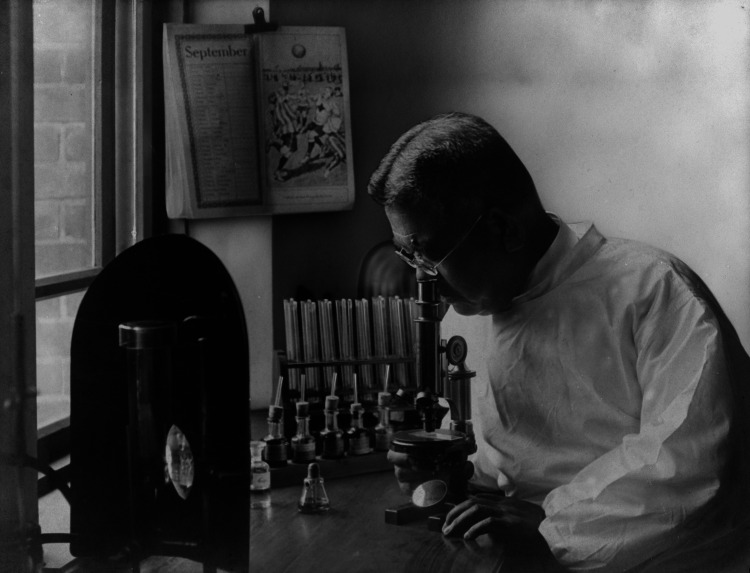
Dr. Shibasaburo Kitasato conducting research in his laboratory in 1910 (at the age of 57 years). Photo courtesy of the Kitasato Institute Shibasaburo Memorial Museum. This image is in the public domain due to copyright expiration.

Figure 2 depicts Dr. Shibasaburo Kitasato deeply engrossed in his research, at the age of 57 years in 1910. The photograph showcases Dr. Kitasato's ongoing commitment to scientific inquiry, nearly two decades after his groundbreaking work on serum therapy. The image depicts Dr. Kitasato using a microscope, surrounded by various laboratory equipment including test tubes and flasks. This setup reflects the advanced state of bacteriological research in the early 20th century, much of which was influenced by Dr. Kitasato's earlier work. The calendar visible in the background, showing the month of September, provides a glimpse into the day-to-day life of this eminent scientist. This photograph not only illustrates Dr. Kitasato's enduring dedication to research but also symbolizes the long-term impact of his earlier discoveries, including serum therapy, which continued to shape medical practices well into the 20th century.

Pioneer of modern medicine in Japan

Kitasato continued to expand his contributions far beyond the creation of a single institution, encompassing the broad spectrum of medical education, research, and healthcare systems in Japan. When the Institute for Infectious Diseases came under government control in 1914, Kitasato, determined to uphold his founding principles, established the Kitasato Institute, which became instrumental in nurturing exceptional researchers and significantly advancing the fields of bacteriology and immunology. His commitment to medical education culminated in the founding of Keio University School of Medicine in 1917, where he served as the first dean, pioneering a new model of medical education that integrated theory with practice [1]. His influence extended to the realm of medical policy and administration when he became the first president of the Japan Medical Association (then known as the Greater Japan Medical Association) in 1916, a position through which he significantly influenced the modernization and standardization of Japan's medical system.

Kitasato's philosophy and teachings

Kitasato's approach to research and medicine was grounded in two core principles as follows: first, the integration of research with clinical practice, and second, the focus on research topics with clear practical value. He believed that the ultimate mission of a physician was not only to treat but to prevent illness. This preventive philosophy, which he outlined in his early speech "Thoughts on the way of medicine," guided much of his work, particularly in the development of serum therapy, where his focus was on practical solutions that could save lives [12].

Kitasato also emphasized the importance of passion and sincerity ("netsu" and "makoto") in scientific endeavors [13]. A notable example of this was when a young Torasaburo Araki, who would later become the president of Kyoto Imperial University, visited Kitasato in Berlin in 1891. Kitasato, then 38 years old, encouraged him with the following words: "With passion and sincerity, anything can be achieved. If there is any obstacle, it is not the world that is at a standstill, but a lack of passion and sincerity in the individual." These words deeply resonated with Araki, who devoted himself to his studies and became a leading figure in biochemistry. Kitasato's belief in the power of dedication and integrity in research continues to influence medical professionals today, emphasizing that the pursuit of scientific knowledge must be driven by both technical precision and ethical responsibility.

Kitasato's legacy

Shibasaburo Kitasato's life and work were instrumental in the development of modern medicine. His pioneering achievements have left an indelible mark on the field of medicine, influencing generations of researchers and medical practitioners well beyond his lifetime. Kitasato's legacy is multifaceted and far-reaching. His work on serum therapy laid the foundation for modern immunology and vaccination strategies. The principles he established continue to guide research in these fields today, contributing to the development of treatments for various diseases and the ongoing fight against global pandemics [14].

In Japan, Kitasato's influence on medical education and research infrastructure is evident in the institutions he founded, such as the Kitasato Institute and the Keio University School of Medicine, which remain at the forefront of medical research and education [15]. His emphasis on practical education and integrating research with clinical practice has shaped the approach to medical training in Japan.

Kitasato also mentored a generation of scientists who made significant contributions to medicine and public health. Among them were Tomoe Takagi (1858-1943), a leader in Japanese surgery, Taiichi Kitajima (1870-1956), a prominent bacteriologist, and Kiyoshi Shiga (1871-1957), who discovered the *Shigella dysenteriae* bacterium. Sahachiro Hata (1873-1938) codeveloped the first effective treatment for syphilis with Paul Ehrlich, while Mikinosuke Miyajima (1872-1944) and Shinkichi Umeno (1862-1930) made advances in bacteriology, immunology, and pathology [16]. Hideyo Noguchi (1876-1928) gained international acclaim for his research on yellow fever [17]. These disciples not only advanced Kitasato’s legacy but also established Japan as a leader in medical research and education, reflecting the lasting impact of his mentorship.

Internationally, Kitasato's collaborative work with researchers like Robert Koch and Emil von Behring helped establish Japan as a significant contributor to global medical research. His achievements encouraged subsequent generations of Japanese researchers to engage in international scientific discourse, fostering a tradition of global collaboration in medical research [5].

Kitasato's philosophy of conducting research with clinical applications in mind has had a lasting impact on the approach to medical research. His emphasis on the practical application of scientific discoveries has influenced the development of translational medicine, a field that aims to "translate" findings from basic science to practical applications that enhance human health and well-being [14].

As we commemorate Kitasato's legacy with his portrait on the new 1000-yen banknote in July 2024, it's an opportune moment for medical professionals to revisit and draw inspiration from his teachings. Kitasato's enduring influence serves as a reminder of the potential for scientific research to profoundly impact society and the importance of pursuing research with both passion and practical purpose.

## Conclusions

Shibasaburo Kitasato's groundbreaking work in bacteriology and immunology, particularly his development of serum therapy, has left an indelible mark on modern medicine. His establishment of research institutions and emphasis on practical medical education significantly advanced Japan's position in global medical research, fostering a legacy of international collaboration. Kitasato's enduring philosophy of conducting research with clinical applications in mind continues to inspire contemporary medical professionals, exemplifying the profound impact scientific inquiry can have on society and serving as a beacon for future generations of researchers.
